# Adaptive Approximation Sliding-Mode Control of an Uncertain Continuum Robot with Input Nonlinearities and Disturbances

**DOI:** 10.1155/2024/8533606

**Published:** 2024-03-06

**Authors:** Shoulin Xu

**Affiliations:** Institute of Logistics Science and Engineering, Shanghai Maritime University, Shanghai, China

## Abstract

This paper develops an adaptive nonsingular fast terminal sliding-mode control (ANFTSMC) scheme for the continuum robot subjected to uncertain dynamics, external disturbances, and input nonlinearities (e.g., actuator deadzones/faults). Concretely, a function approximation technique (FAT) is utilized to estimate unknown robot dynamics and actuator deadzones/faults online. Furthermore, a disturbance observer (DO) is devised to make up for unknown external disturbances. Then, an ANFTSMC scheme combined with FAT and DO is developed, to expedite the restoration of the stability for the continuum robot. The proposed ANFTSMC not only can retain the benefits of traditional terminal sliding-mode control (TSMC), containing easy enforcement, quick response, and robustness to uncertainties but also dispose of the latent singularity for traditional faster TSMC designs. Afterward, the simulation results show that the proposed controller can effectively improve the trajectory tracking accuracy of the continuum robot, and the tracking root-mean-square errors are 0.0115 and 0.0128 rad. Finally, the effectiveness of ANFTSMC scheme is validated by experiments.

## 1. Introduction

Continuum robot is a new type of bionic robot, inspired by the natural organisms, which could attune to the sophisticated environments, such as the aircraft assembly, nuclear reactor maintenance, minimally invasive surgery, and so on [1–3]. With the increasing requirements for high precision, safety, and stability, the trajectory tracking control of the continuum robots has always been a topic of the latest research. Nevertheless, compared to the classical rigid robots, the dynamics of the continuum robots is a strongly coupled nonlinear system [4]. Consequently, it is difficult to acquire the accurate dynamics model of the continuum robots [5]. Additionally, in practice, owing to the compliance of the continuum robots, the tracking performance of the continuum robots will be seriously affected by external disturbances and input nonlinearities (e.g., actuator deadzones/faults). Therefore, how to elevate the tracking performance and transient response for uncertain continuum robots, notably in the existence of the external disturbances and actuator deadzones/faults, is still a challenging task for the practical applications [6].

For the uncertainties, several progressive control methods have been developed, such as adaptive control [7], fault tolerant control [8], neural network (NN) control [9], fuzzy control [10], and sliding-mode control (SMC) [11]. Among them, SMC is an efficacious robust technology, resulting from its strong robustness to resist uncertainties [12–14]. Nevertheless, the conventional SMC is poor in treating the rapid variations of disturbances and is prone to causing chattering [15, 16]. To retain the merits and reduce the weakness of SMC, a terminal SMC (TSMC) has been developed [17, 18]. However, the conventional TSMC has a deficiency in providing lag convergence speed and singularity issues [19]. To handle these problems, the fast TSMC and nonsingular TSMC have been presented separately [20]. Unfortunately, the fast TSMC and nonsingular TSMC do not possess good ability to repress the chattering [21]. Furthermore, most of the available outcomes of TSMC rest with prior information of uncertainties. Nevertheless, the prior information is ordinarily difficult to attain in practical systems [22]. In response to these, some approximation approaches have been presented to approximate uncertainties and restrain the chattering without requiring prior knowledge. Thus, it is necessary to develop efficacious approximation methods to estimate uncertainties [23].

Recently, the approximation capability of fuzzy logic system (FLS) and NN has been popularly operated to approximate unknown functions [24, 25]. A novel adaptive control scheme was presented to assure that the nonlinear system is stable by FLS [26]. To improve the robustness, an adaptive constrained controller using FLS was proposed for the flight vehicles [27]. A NN-based adaptive attitude controller was designed for the spacecraft [28]. Moreover, an adaptive switching control method using NN was proposed for a nonlinear system [29]. However, it is worth noting that FLS and NN methods mainly have good approximation ability to continuous functions, but poor approximation capability to discontinuous functions. Furthermore, they commonly offer large approximation errors and poor approximation performance for unknown external disturbances.

To tackle the unknown disturbances, the disturbance observer (DO) is an efficacious solution and raises robustness [30, 31]. Considering the input lag, a nonlinear DO was used to estimate the disturbance for uncertain systems, which increased the stability of the systems [32]. Furthermore, a DO-based bound adaptive controller was proposed to restrain the deflection of flexible manipulator, fulfill angular positioning, and obstruct unknown disturbances [33]. A DO was designed to mitigate the impact of uncertain disturbances for robotic systems [34]. To enhance accuracy, a DO was developed to evaluate disturbances, which was incorporated in integral SMC to set-off disturbances [35]. To increase the ability of antidisturbance, a hierarchical dynamic surface control method was designed for the singularly perturbed systems based on dual DO [36]. A robust control scheme utilizing a finite-time DO was proposed for flexible-joint robots to achieve the trajectory tracking and disturbance suppression [37]. A robust DO-based controller was designed to guarantee the prolonged stability for the systems [38]. Nevertheless, to the author's knowledge, these aforementioned DO methods can only assure the asymptotic convergence of estimation errors, which will ultimately impact the settling time of the system states.

However, as discussions mentioned above, how to actualize the excellent tracking control for the continuum robot is still an open problem, notably in the existence of the uncertainties, disturbances, and actuator deadzones/faults. Here, some problems in continuum robot systems are summarized as follows: (1) FLS and NN methods have good approximation ability to continuous function, but poor approximation ability to discontinuous function. However, the uncertain dynamics and actuator deadzones/faults of the continuum robot may be discontinuous function, so how to achieve good approximation ability of discontinuous function is a work worthy of further study. (2) Ordinary DOs are unsuited for uncertain robot systems, as most DOs are structured based on known robot system information. Consequently, how to fulfill effective real-time estimation of unknown external disturbances is a challenge. (3) Although TSMC method has good robustness against uncertainties, the better the robustness, the worse the control accuracy of the continuum robot may be. Thus, it is necessary to design a hybrid controller that can combine the advantages of TSMC, approximation methods, and DO. Unfortunately, there are few results in the reported literature to fulfill this amusing control method. The reason may be that it is difficult to reestablish the control inputs of the hybrid system to assure the stability and convergence. Furthermore, so far, the reported literature on TSMC using approximation method and DO for the continuum robot simultaneously suffered from uncertain dynamics, disturbances, and actuator deadzones/faults are scarce. This is the motivation of this paper.

In this paper, a new adaptive nonsingular fast terminal sliding-mode control (ANFTSMC) scheme combined with function approximation technique (FAT), and DO is proposed for the continuum robot subjected to uncertain dynamics, external disturbances, and actuator deadzones/faults. The main contributions are summarized as follows:

1. A FAT is introduced to estimate uncertain dynamics and actuator deadzones/faults. More importantly, the proposed FAT effectively solves the approximation problem of continuous and discontinuous functions in robot systems.
2. A DO is proposed to estimate the external disturbances without the system information, which can eliminate the external disturbances quickly and provide higher estimation accuracy in real-time.
3. An ANFTSMC combined with FAT and DO is proposed for uncertain continuum robot, and simulation and experiment verification show that proposed ANFTSMC scheme effectively achieves faster convergence speed and higher tracking accuracy for the continuum robot.

## 2. System Description and Problem Formulation

### 2.1. Robot Dynamics Model

**Figure 1:**
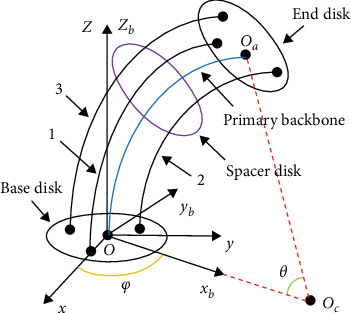
The bending model of the continuum robot.

The continuum robot is constituted of a base disk, several spacer disks, an end disk, and four resilient NiTi wires. The middle NiTi wire denotes primary backbone, and the rest NiTi wires express secondary backbones [2]. The simplified bending model of the continuum robot is assumed to be a circular arc, as shown in Figure 1 [5]. Point *O* is the central point of base disk, and the global coordinate system *O* − *xyz* and bending coordinate system *O* − *x*_*b*_*y*_*b*_*z*_*b*_ are set up, respectively. *i*(*i* = 1, 2, 3) denotes the label of secondary backbone. Angle *θ* denotes the angle of bending primary backbone in *O* − *x*_*b*_*z*_*b*_ plane. Angle *φ* is the angle between the axis *x* and *x*_*b*_. Secondary backbones revolve around primary backbone of the mean intermission in 120°. *r* denotes the distance from primary backbone to every secondary backbone. *L* expresses the length of primary backbone.

The dynamics of the continuum robot are described by the following equation [3, 5]:

(1) $$\begin{array}{c} M_{0}\left(q\right)\ddot{q}+C_{0}\left(q,\dot{q}\right)\dot{q}+G_{0}\left(q\right)+\Delta H\left(q,\dot{q},\ddot{q}\right)+\delta =F, \end{array}$$

where *q* = [*θ*, *φ*]^*T*^ expresses the vector of the joint variable, *M*_0_(*q*) ∈ *R*^2×2^ stands for the nominal inertia matrix, $C_{0}\left(q,\dot{q}\right)\in R^{2\times 2}$ denotes the nominal matrix of Coriolis and centripetal forces, *G*_0_(*q*) ∈ *R*^2^ is the nominal gravity vector, *δ* ∈ *R*^2^ expresses the external disturbance, *F* = [*f*_1_, *f*_2_]^*T*^ stands for the driving force. $\Delta H\left(q,\dot{q},\ddot{q}\right)$ expresses the uncertain term in dynamics as follows:

(2) $$\begin{array}{c} \Delta H\left(q,\dot{q},\ddot{q}\right)=\Delta M\left(q\right)\ddot{q}+\Delta C\left(q,\dot{q}\right)\dot{q}+\Delta G\left(q\right)+F_{b}\left(q,\dot{q}\right), \end{array}$$

where Δ*M*(*q*) ∈ *R*^2×2^ denotes uncertain inertia matrix, $\Delta C\left(q,\dot{q}\right)\in R^{2\times 2}$ stands for uncertain Coriolis and centrifugal matrix, Δ*G*(*q*) ∈ *R*^2^ expresses uncertain gravity vector, and $F_{b}\left(q,\dot{q}\right)\in R^{2}$denotes friction.

**Remark 1 .**

Owing to the fact that the continuum robot possesses 2° of freedom, the configuration of the continuum robot can be decided by two driving forces. Furthermore, the framework of the continuum robot is symmetrical about the center of primary backbone, so the driving forces *f*_1_, *f*_2_, and *f*_3_ have the properties of spatial center symmetric about primary backbone. The continuum robot can be driven by pulling any two independent secondary backbones among three secondary backbones. Here, Equation (1) is based on the assumption that *f*_1_ and *f*_2_ are driving forces.

**Property 1 .**

Matrix *M*_0_(*q*) is symmetric and positive definite, and matrix $\left(1/2\right){\dot{M}}_{0}\left(q\right)-C_{0}\left(q,\dot{q}\right)$ is skew-symmetric [9].

The model of actuator deadzone is described as follows [23]:

(3) $$\begin{array}{c} F_{\text{di}}=\left\{\begin{array}{c} d_{\text{mi}}\left(u_{\text{Fi}}-b_{\text{mi}}\right),\\ 0,\begin{array}{ccc} \; & \; & \; \end{array}\\ d_{\text{Mi}}\left(u_{\text{Fi}}-b_{\text{Mi}}\right), \end{array}\right.\begin{array}{c} u_{\text{Fi}}\leq b_{\text{mi}}\\ b_{\text{mi}}<u_{\text{Fi}}<b_{\text{Mi}}\\ u_{\text{Fi}}\geq b_{\text{Mi}}, \end{array} \end{array}$$

where *u*_Fi_ expresses the control input force, *i* = 1, 2. *d*_Mi_ and *d*_mi_ denote deadzone parameters, and *b*_Mi_ and *b*_mi_ represent upper and lower deadzone bounds, respectively.

Furthermore, the actuator fault is expressed by the following equation [18]:

(4) $$\begin{array}{c} F_{\text{ai}}=\sigma_{i}u_{\text{Fi}}+\phi_{i}\left(t\right),i=1,2, \end{array}$$

where *σ*_*i*_ ∈ (0, 1) expresses an unknown gain factor for the *i*th actuator joint, and *ϕ*_*i*_(*t*) stands for the bias force.

From Equations (3) and (4), the control force of the continuum robot can be denoted by the following equation:

(5) $$\begin{array}{c} F=F_{d}+F_{a}=K_{u}u_{F}+\Delta u_{F}, \end{array}$$

where *u*_*F*_ = [*u*_*F*1_, *u*_*F*2_]^*T*^ is the control input force, *K*_*u*_ = diag[*k*_*u*1_, *k*_*u*2_] expresses a known matrix, and Δ*u*_*F*_ expresses the unknown part.

Define $x_{1}≜q,x_{2}≜\dot{q},u≜u_{F}$, substituting Equation (5) into Equation (1), then, the dynamics of Equation (1) can be rewritten as follows:

(6) $$\begin{array}{c} \left\{\begin{array}{c} {\dot{x}}_{1}=x_{2}\begin{array}{cccc} \; & \; & \; & \; \end{array}\begin{array}{cccc} \; & \; & \; & \; \end{array}\begin{array}{cccc} \; & \; & \; & \; \end{array}\begin{array}{cccc} \; & \; & \; & \; \end{array}\\ {\dot{x}}_{2}=M_{0}^{-1}\left(x_{1}\right)\left(u-C_{0}\left(x_{1},x_{2}\right)x_{2}-G_{0}\left(x_{1}\right)-\gamma \left(x_{1},x_{2},u\right)-\delta \right) \end{array}\right., \end{array}$$

where $\gamma \left(x_{1},x_{2},u\right)=\left(I-K_{u}\right)u-\Delta u+\Delta H\left(x_{1},x_{2},{\dot{x}}_{2}\right)$ expresses a new lumped unknown term, and *I* ∈ *R*^2×2^ is an identity matrix.

**Lemma 1 .**

For ∀*ς*_*i*_ ∈ *R*, the following inequality holds:

(7) $$\begin{array}{c} {\left(\sum_{i=1}^{n}\left|\zeta_{i}\right|\right)}^{p_{a}}\leq \sum_{i=1}^{n}{\left|\zeta_{i}\right|}^{p_{a}}\leq n^{1-p}{\left(\sum_{i=1}^{n}\left|\zeta_{i}\right|\right)}^{p_{a}}, \end{array}$$

where 0 < *p*_*a*_ ≤ 1, *i* = 1, 2,…, *n* [7, 11].

**Lemma 2 .**

For ∀*χ*_1_, *χ*_2_ ∈ *R*, and constants *ϑ*_1_ > 0, *ϑ*_2_ > 0, and *ϑ*_3_ > 0, one has the following equation [9, 12]:

(8) $$\begin{array}{c} {\left|\chi_{1}\right|}^{\vartheta_{1}}{\left|\chi_{2}\right|}^{\vartheta_{2}}\leq \frac{\vartheta_{1}\vartheta_{3}}{\vartheta_{1}+\vartheta_{2}}{\left|\chi_{1}\right|}^{\vartheta_{1}+\vartheta_{2}}+\frac{\vartheta_{2}\vartheta_{3}}{\vartheta_{1}+\vartheta_{2}}{\left|\chi_{2}\right|}^{\vartheta_{1}+\vartheta_{2}}. \end{array}$$

**Lemma 3 .**

If there exists a continuous and positive functions *V*(*x*) > 0, satisfying the following equation:

(9) $$\begin{array}{c} \dot{V}\left(x\right)\leq -c_{1}V\left(x\right)+c_{2}, \end{array}$$

where *c*_1_ and *c*_2_ are positive constants. Then, the solution *x*(*t*) is uniformly stable [15].

**Remark 2 .**

*ς* = (*ς*_1_, *ς*_2_,…,*ς*_*n*_)^*T*^ denotes a *n*-dimensional vector, and *α* is a positive constant; then, the symbols are defined as follows:

(10) $$\begin{array}{c} \varsigma^{\alpha }={\left(\varsigma_{1}^{\alpha },\varsigma_{2}^{\alpha },\ldots ,\varsigma_{n}^{\alpha }\right)}^{T}, \end{array}$$

(11) $$\begin{array}{c} {\left[\varsigma \right]}^{\alpha }=\text{diag}\left[\varsigma_{1}^{\alpha },\varsigma_{2}^{\alpha },\ldots ,\varsigma_{n}^{\alpha }\right], \end{array}$$

(12) $$\begin{array}{c} \left\|\varsigma \right\|={\left(\varsigma_{1}^{2}+\varsigma_{2}^{2}+\cdots +\varsigma_{n}^{2}\right)}^{1/2}. \end{array}$$

**Assumption 1.**

The disturbance *δ* is continuous and bounded, satisfying ‖*δ*‖ ≤ *b*_1_, where *b*_1_ > 0 is an unknown constant [30].

**Assumption 2.**

The derivative of *δ* is unknown and bounded, satisfying $\left\|\dot{\delta }\right\|\leq h_{1}$, where *h*_1_ > 0 is an unknown constant [30, 32].

**Remark 3 .**

In actual robot systems, the disturbance that robots are subjected to is bounded, but the exact bounded value of the disturbance is unknown. In addition, the derivative of the disturbance is bounded, but the exact bounded value of the derivative for the disturbance is unknown.

### 2.2. Function Approximation Technique Using Fourier Series Expansion

According to the Stone–Weierstrass theorem, the orthogonal basis functions offer a universal function approximator for arbitrary nonlinear systems with random precision [39, 40]. The definition of orthogonal basis function is described as follows:

**Definition 1 .**

An inner product is given by the following equation:

(13) $$\begin{array}{c} \langle f\left(x\right),g\left(x\right)\rangle =\int \hat{f}\left(x\right)g\left(x\right)dx, \end{array}$$

where $\hat{f}\left(x\right)$ denotes the complex conjugate of function *f*(*x*).

If the inner product of function in Equation (13) is equal to zero with *f*(*x*) ≠ *g*(*x*), then, *f*(*x*) and *g*(*x*) are called the orthogonal functions.

**Lemma 4 .**

For any real-valued periodic or aperiodic function *f*(*x*), which satisfies the Dirichlet conditions, then *f*(*x*) can be represented as the sum of its FS over a time interval [5, 40].

If function *g*(*t*) satisfies Dirichlet conditions in the time interval [*t*_1_, *t*_2_], then *g*(*t*) can be denoted by the following equation [41]:

(14) $$\begin{array}{c} g\left(t\right)=a_{0}+\sum_{i=1}^{\infty }\left(a_{i}\cos\left(\omega_{i}t\right)+b_{i}\sin\left(\omega_{i}t\right)\right), \end{array}$$

where *a*_0_, *a*_*i*_, and *b*_*i*_ express the FS coefficients of *g*(*t*), *ω*_*i*_ = 2*iπ*/*T* denotes the frequency of sine and cosine functions, and *T* represents the base period of *g*(*t*).

Supposing that function *g*_*n*_(*t*) is expressed as follows:

(15) $$\begin{array}{c} g_{n}\left(t\right)=a_{0}+\sum_{i=1}^{n}\left(a_{i}\cos\left(\omega_{i}t\right)+b_{i}\sin\left(\omega_{i}t\right)\right). \end{array}$$

Then, function *g*_*n*_(*t*) denotes the FS approximation of *g*(*t*), and error is denoted as follows:

(16) $$\begin{array}{c} \varepsilon_{n}=g\left(t\right)-g_{n}\left(t\right)=\sum_{i=n+1}^{\infty }\left(a_{i}\cos\left(\omega_{i}t\right)+b_{i}\sin\left(\omega_{i}t\right)\right). \end{array}$$

Furthermore, function *g*_*n*_(*t*) can be described by the following equation:

(17) $$\begin{array}{c} g_{n}\left(t\right)=W\varphi \left(t\right), \end{array}$$

where

(18) $$\begin{array}{c} W=\left[a_{0},a_{1},b_{1},\ldots ,a_{n},b_{n}\right], \end{array}$$

and

(19) $$\begin{array}{c} \varphi \left(t\right)={\left[1,\cos\left(\omega_{1}t\right),\sin\left(\omega_{1}t\right),\ldots ,\cos\left(\omega_{n}t\right),\sin\left(\omega_{n}t\right)\right]}^{T}. \end{array}$$

## 3. Design of ANFTSMC

This section works out a novel ANFTSMC scheme combined with FAT and DO of the continuum robot.

### 3.1. Controller Design

It is assumed that *x*_1_^*d*^ is the desired trajectory of the continuum robot, and $x_{2}^{d}={\dot{x}}_{1}^{d}$. Furthermore, since the position and velocity of the continuum robot are generally bounded in actual robot operations, it is supposed that *x*_1_^*d*^ and *x*_2_^*d*^ hold $\left|x_{i}^{d}\right|<\overset{―}{x},i=1,2$, and $\overset{―}{x}>0$ is a constant.

The error variables are defined by the following equations:

(20) $$\begin{array}{c} e_{1}=x_{1}-x_{1}^{d}, \end{array}$$

(21) $$\begin{array}{c} e_{2}=x_{2}-\mu_{1}, \end{array}$$

where *e*_1_ = [*e*_11_, *e*_12_]^*T*^, *μ*_1_ = −*K*_*a*_*e*_1_ + *x*_2_^*d*^ denotes a virtual controller, and *K*_*a*_ = diag[*k*_*a*1_, *k*_*a*2_] expresses a positive definite matrix.

From Equations (20) and (21), one has the following equation:

(22) $$\begin{array}{c} {\dot{e}}_{1}=-K_{a}e_{1}+e_{2}. \end{array}$$

Furthermore, the derivative of *e*_2_ is as follows:

(23) $$\begin{array}{c} {\dot{e}}_{2}={\dot{x}}_{2}-{\dot{\mu }}_{1}=M_{0}^{-1}\left(x_{1}\right)\left(u-C_{0}\left(x_{1},x_{2}\right)x_{2}-G_{0}\left(x_{1}\right)-\gamma \left(x_{1},x_{2},u\right)-\delta \right)-{\dot{\mu }}_{1}. \end{array}$$

The choice of sliding surfaces possesses a momentous influence on the performance of the continuum robot systems. The selection of sliding surfaces enables it to meet with the expected performance of the system when it converges to zero. In order to fulfill a fast transient response convergence without singularity issue, an ANFTSMC surface is selected by the following equation [12, 19]:

(24) $$\begin{array}{c} s=K_{0}e_{1}+K_{1}e_{1}^{\lambda_{1}}+K_{2}e_{2}^{p_{1}/q_{1}}, \end{array}$$

where *s* expresses the sliding surfaces variable. *K*_0_ = diag[*k*_01_, *k*_02_], *K*_1_ = diag[*k*_11_, *k*_12_], and *K*_2_ = diag[*k*_21_, *k*_22_] represent positive definite matrices, respectively. *p*_1_ and *q*_1_ denote positive odd numbers holding 1 < *p*_1_/*q*_1_ < 2 and *λ*_1_ > *p*_1_/*q*_1_.

The derivative of *s* in Equation (24) can be yielded as follows:

(25) $$\begin{array}{c} \dot{s}=K_{0}{\dot{e}}_{1}+K_{1}\lambda_{1}{\left[e_{1}\right]}^{\lambda_{1}-1}{\dot{e}}_{1}+K_{2}\frac{p_{1}}{q_{1}}{\left[e_{2}\right]}^{\left(p_{1}/q_{1}-1\right)}{\dot{e}}_{2}. \end{array}$$

Furthermore, in the light of FAT, the lumped unknown term *γ*(*x*_1_, *x*_2_, *u*) can be represented by the following equation [40, 41]:

(26) $$\begin{array}{c} \gamma \left(x_{1},x_{2},u\right)=W^{*T}\varphi \left(Z\right)+\varepsilon_{Z}, \end{array}$$

where *W*^*∗*^ = diag[*w*_1_^*∗*^, *w*_2_^*∗*^] expresses the ideal weight matrix, and *w*_*i*_^*∗*^ = [*w*_*i*1_^*∗*^, *w*_*i*2_^*∗*^,…,*w*_in_^*∗*^]^*T*^. *Z* = [*x*_1,_, *x*_2_, *u*]^*T*^ stands for the input variable, *φ*(*Z*) = [*φ*_1_(*Z*), *φ*_2_(*Z*)]^*T*^ is the basis function, and *φ*_*i*_(*Z*) = [*φ*_*i*1_(*Z*), *φ*_*i*2_(*Z*),…,*φ*_in_(*Z*)]^*T*^. *ε*_*Z*_ represents the estimation error, and |*ε*_*Z*_| < *ε*.

The adaption law of FAT is designed by the following equation:

(27) $$\begin{array}{c} {\dot{\hat{w}}}_{i}=-\Gamma_{i}\left(s_{i}k_{2i}\frac{p_{1}}{q_{1}}\varphi_{i}\left(Z\right)e_{2i}^{\left(p_{1}/q_{1}\right)-1}+\xi_{i}{\hat{w}}_{i}\right), \end{array}$$

where ${\hat{w}}_{i}\left(i=1,2\right)$ expresses the actual weight utilized to estimate ideal weight *w*_*i*_^*∗*^, Γ_*i*_ denotes a positive constant, and *ξ*_*i*_ represents a small positive constant.

**Remark 4 .**

It is noted that FLS and NN methods have a good approximation performance to continuous functions, but poor approximation ability to discontinuous functions [27, 29]. However, in practical applications for the continuum robot, the abrupt and intermittent actuator deadzones/faults may be discontinuous. Aiming these situations, FLS and NN will offer a large estimation error and poor control performance. In contrast with FLS and NN approaches, the proposed FAT possesses a good capability to approximate continuous and discontinuous unknown functions, respectively. Therefore, in this paper, FAT is utilized to estimate the unknown function *γ*(*x*_1_, *x*_2_, *u*), which integrates the unknown dynamics, frictions, and actuator deadzones/faults of the continuum robot.

In the light of Assumption 1, the upper bound of the external disturbance is unknown. Define the DO as $\hat{\delta }$. Then, the estimation error $\tilde{\delta }$ is given by the following equation:

(28) $$\begin{array}{c} \tilde{\delta }=\hat{\delta }-\delta . \end{array}$$

The time derivative of Equation (28), one has the following equation:

(29) $$\begin{array}{c} \dot{\tilde{\delta }}=\dot{\hat{\delta }}-\dot{\delta }. \end{array}$$

The adaption law of DO is designed as follows:

(30) $$\begin{array}{c} {\dot{\hat{\delta }}}_{i}=-\Lambda_{i}\left(s_{i}K_{2i}\frac{p_{1}}{q_{1}}e_{2i}^{\left(p_{1}/q_{1}\right)-1}+\eta_{i}{\hat{\delta }}_{i}\right), \end{array}$$

where Λ_*i*_ and *η*_*i*_ denote two positive constants, *i* = 1, 2.

**Remark 5 .**

In practical robot systems, it is often difficult or even impossible to physically measure external disturbances. Nevertheless, common DOs are no longer suitable for uncertain robot systems, as most DOs are constructed based on known system information. Consequently, in contrast with the conventional DO-based control approaches, a DO is introduced to estimate the unknown external disturbance *δ*. The advantage of the proposed DO is that it can quickly obviate the influence of external disturbance and provide higher estimation precision. In addition, another benefit is that the proposed control law of the continuum robot contains the estimation $\hat{\delta }$. This helps to estimate unknown external disturbances in real-time and automatically update the control law based on the uncertainty. Therefore, the tracking performance of the continuum robot system can be greatly improved.

The ANFTSMC scheme is proposed by the following equation:

(31) $$\begin{array}{c} u=u_{1}+u_{2}, \end{array}$$

where

(32) $$\begin{array}{c} u_{1}=C_{0}\left(x_{1},x_{2}\right)x_{2}+G_{0}\left(x_{1}\right)+M_{0}\left(x_{1}\right){\dot{\mu }}_{1}, \end{array}$$

(33) $$\begin{array}{c} \begin{array}{ccc} u_{2}= & - & \frac{q_{1}}{p_{1}}K_{2}^{-1}{\left[e_{2}\right]}^{1-\left(p_{1}/q_{1}\right)}M_{0}\left(x_{1}\right)\left(K_{0}{\dot{e}}_{1}+K_{1}\lambda_{1}{\left[e_{1}\right]}^{\lambda_{1}-1}{\dot{e}}_{1}\right)\\ \; & - & \frac{q_{1}}{p_{1}}K_{2}^{-1}{\left[e_{2}\right]}^{1-\left(p_{1}/q_{1}\right)}\left(C_{0}\left(x_{1},x_{2}\right)s+\beta s\text{sgn}\left(s\right)+\frac{s}{{\left\|s\right\|}^{2}}e_{1}^{T}e_{2}\right)\\ \; & + & {\hat{W}}^{T}\varphi \left(Z\right)+\hat{\delta }, \end{array} \end{array}$$

where *β* is a positive constant.

### 3.2. Stability Analysis

**Theorem 1 .**

Consider the continuum robot system (Equation (6)) in the presence of the uncertain dynamics, external disturbances, and actuator deadzones/faults. If the proposed ANFTSMC scheme (Equations (31)–(33)), combined with FAT (Equation (26)), FAT adaption law (Equation (27)), and DO adaption law (Equation (30)), is employed for the continuum robot system (Equation (6)), then the error variables $e_{1},s,{\tilde{w}}_{i}$, and $\tilde{\delta }$ are uniformly bounded.

The proof of Theorem 1 is given in the Appendix.

**Remark 6 .**

The proposed sliding surface *s* effectually combines the features of FTSMC and NTSMC methods [34], enabling the continuum robot system to fulfill the fast finite-time convergence without singularity difficulty. Furthermore, the proposed ANFTSMC in Equations (31)–(33) can increase the transient response speed and diminish the steady state error of the continuum robot system. When *s* converges to zero, then, one has the error *e*_1_ = 0. The proposed control method still has chattering, which will affect the control performance of the continuum robot. The chattering of the continuum robot can be alleviated by adjusting the parameters of the proposed ANFTSMC, and a balance selection can be made between tracking accuracy and chattering.

## 4. Simulation Results

To verify the effectiveness of the proposed ANFTSMC scheme, the simulations are performed on a continuum robot, whose parameters are designed as *L* = 210 mm and *r* = 5 mm. The dynamics model of the continuum robot can be derived based on Lagrange dynamics approach [2]. Furthermore, to simplify simulation, only secondary backbones 1 and 2 of the continuum robot generate driving forces, which make the continuum robot bend. The sampling period is set by 0.5 ms.

The initial position and velocity of the continuum robot are set as *q*(0) = [0, 0]^*T*^ rad and $\dot{q}\left(0\right)={\left[0,0\right]}^{T}$ rad/s, respectively. The desired trajectory *q*_*d*_ = [*θ*_*d*_, *φ*_*d*_]^*T*^ is selected as follows:

(34) $$\begin{array}{c} \theta_{d}=0.9-0.9\cos\left(\frac{\pi }{36}t\right), \end{array}$$

(35) $$\begin{array}{c} \varphi_{d}=\frac{2\pi }{3}\sin\left(\frac{\pi }{48}t\right). \end{array}$$

The friction is chosen by the following equation:

(36) $$\begin{array}{c} F_{b}\left(q,\dot{q}\right)=\left[\begin{array}{c} 0.5\dot{\theta }-0.2\;\sin\left(5\varphi \right)+0.6\;\cos\left(2\theta \right)\\ 1.2\dot{\varphi }+0.8\;\cos\left(3\dot{\theta }\right)+0.3\;\sin\left(6\theta \right) \end{array}\right]. \end{array}$$

Here, the external disturbance is selected as follows:

(37) $$\begin{array}{c} \delta =\left[\begin{array}{c} 3\;\sin t-5\;\cos t+2\;\cos^{2}t\\ 2\;\sin^{2}t+6\;\sin t-5\;\cos t \end{array}\right]. \end{array}$$

The parameters of actuator deadzones in Equation (3) are *d*_*m*1_ = *d*_*m*2_ = 0.6, *d*_*M*1_ = *d*_*M*2_ = 0.8, *b*_*m*1_ = *b*_*m*2_ = 2.5, and *b*_*M*1_ = *b*_*M*2_ = 3.2, respectively. Furthermore, abrupt actuator faults are considered only, as their impact on the continuum robot system is much larger than that incipient actuator faults. The parameters of actuator faults in Equation (4) are selected by the following equations:

(38) $$\begin{array}{c} \sigma_{1}=\left\{\begin{array}{c} 1,\begin{array}{cc} \; & \; \end{array}\text{ if }t<8s\\ 0.7+0.1\;\sin t,\text{ if }t\geq 8s \end{array}\right.,\;\phi_{1}\left(t\right)=\left\{\begin{array}{c} 0,\text{ if }t<8s\\ -0.6,\text{ if }t\geq 8s \end{array}\right., \end{array}$$

(39) $$\begin{array}{c} \sigma_{2}=\left\{\begin{array}{c} 1,\begin{array}{cc} \; & \; \end{array}\text{ if }t<8s\\ 0.5+0.2\;\cos t,\text{ if }t\geq 8s \end{array}\right.,\;\phi_{2}\left(t\right)=\left\{\begin{array}{c} 0,\text{ if }t<8s\\ -0.8,\text{ if }t\geq 8s \end{array}\right.. \end{array}$$

In Equations (38) and (39), it means that the abrupt actuator faults occur in *t* = 8 s, that is, actuator faults are discontinuous. Furthermore, the initial weight matrix of FS is selected as ${\hat{w}}_{i}\left(0\right)=0\in R^{15}\left(i=1,2\right)$, and basis function of FS is chosen as follows:

(40) $$\begin{array}{c} \varphi_{i}\left(Z\right)={\left[1,\cos\;\left(\omega_{1}t\right),\sin\;\left(\omega_{1}t\right),\cos\;\left(2\omega_{1}t\right),\sin\;\left(2\omega_{1}t\right),\cos\;\left(3\omega_{1}t\right),\sin\;\left(3\omega_{1}t\right),\ldots ,\cos\;\left(7\omega_{1}t\right),\sin\;\left(7\omega_{1}t\right)\right]}^{T}\in R^{15}, \end{array}$$

where parameter *ω*_1_ is set as *π*/8.

To manifest the merits of the proposed ANFTSMC for the continuum robot, comparative simulations with existing well-known advanced controllers, such as TSMC [19], and adaptive fuzzy SMC (AFSMC) are conducted [15].

The control input of TSMC is designed by the following equation:

(41) $$\begin{array}{c} u_{F}=M_{0}\left(q\right)\left[{\ddot{q}}_{d}-K_{\sigma }s-K_{\eta }{\left|s\right|}^{q_{a}}\text{Sgn}\left(s\right)\right]-\frac{M_{0}\left(q\right)}{\lambda_{a}p_{a}}{\text{Sig}}^{2-p_{a}}\left({\dot{e}}_{1}\right), \end{array}$$

with

(42) $$\begin{array}{c} s=e_{1}+\lambda_{a}{\left|e_{2}\right|}^{p_{a}}\text{Sgn}\left(e_{2}\right), \end{array}$$

where *K*_*σ*_ ∈ *R*^2×2^ and *K*_*η*_ ∈ *R*^2×2^ express diagonal positive definite matrices, *λ*_*a*_ > 0, 1 < *p*_*a*_ < 2, and 0 < *q*_*a*_ < 1.

The control input of AFSMC is given as follows:

(43) $$\begin{array}{c} u_{F}=\frac{\hat{M}\left(q\right)}{\lambda_{1}}\left(\lambda_{1}{\ddot{q}}_{d}+\lambda_{1}\hat{R}\left(q,\dot{q}\right)-\lambda_{0}e_{2}-\lambda_{2}e_{1}-K_{\beta }s-K_{\omega }\text{Sgn}\left(s\right)\right), \end{array}$$

with

(44) $$\begin{array}{c} s=\hbar_{0}e_{1}+\hbar_{1}e_{2}+\hbar_{2}\int_{0}^{t}e_{1},R\left(q,\dot{q}\right)=-\dot{M}\left(q\right)\left(C\left(q,\dot{q}\right)\dot{q}+G\left(q\right)+F_{b}\right), \end{array}$$

(45) $$\begin{array}{c} \hat{M}\left(q\right)={\hat{\theta }}_{M}^{T}\phi \left(q,\dot{q}\right),\hat{R}\left(q,\dot{q}\right)={\hat{\theta }}_{R}^{T}\phi \left(q,\dot{q}\right), \end{array}$$

(46) $$\begin{array}{c} {\dot{\hat{\theta }}}_{M_{i}}=\xi_{M}\lambda_{1}s_{i}\phi_{i}\left(q,\dot{q}\right)u_{F_{i}}\;{\dot{\hat{\theta }}}_{R_{i}}=\xi_{R}\lambda_{1}\phi_{i}\left(q,\dot{q}\right), \end{array}$$

where *ħ*_0_, *ħ*_1_, *ħ*_2_, *ξ*_*M*_, and *ξ*_*R*_ are positive constants. *K*_*β*_ ∈ *R*^2×2^ and *K*_*ω*_ ∈ *R*^2×2^ denote diagonal positive definite matrices. *θ*_*M*_ and *θ*_*R*_ are the weight vector. $\phi \left(q,\dot{q}\right)$ is the basis function vector. *θ*_*M*_*i*__, *θ*_*R*_*i*__, *s*_*i*_, and $\phi_{i}\left(q,\dot{q}\right)$ are the *i*(*i* = 1, 2)th component of *θ*_*M*_, *θ*_*R*_, *s*, and $\phi \left(q,\dot{q}\right)$, respectively.

Furthermore, the parameters utilized in the above controllers are provided based on the trial and error, up to good tracking accuracy is fulfilled, and are summed up in Table 1.

**Figure 2:**
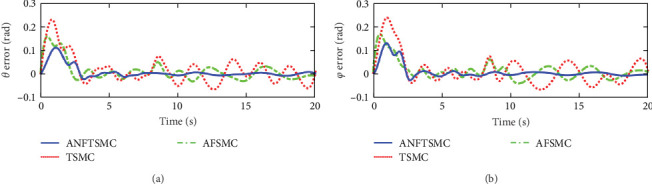
Simulations of the tracking errors: (a) tracking errors of *θ* and (b) tracking errors of *φ*.

**Figure 3:**
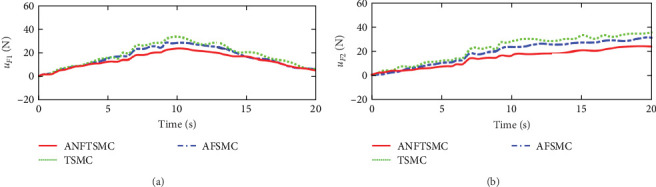
Simulations of the driving forces: (a) driving force *u*_*F*1_ and (b) driving force *u*_*F*2_.

**Figure 4:**
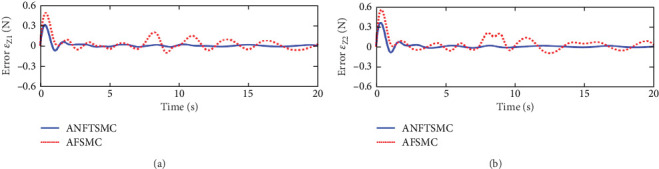
Error *ε*_*Z*_ = [*ε*_*Z*1_, *ε*_*Z*2_]^*T*^ of ANFTSMC scheme and AFSMC method: (a) error *ε*_*Z*1_ and (b) error *ε*_*Z*2_.

**Figure 5:**
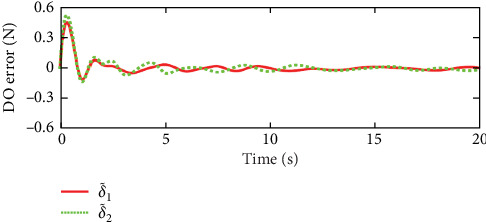
Disturbance error $\tilde{\delta }={\left[{\tilde{\delta }}_{1},{\tilde{\delta }}_{2}\right]}^{T}$ of ANFTSMC scheme.

Figures 2 and 3 illustrate the simulation results of the tracking errors and control inputs of the continuum robot in the existence of the actuator deadzones, actuator faults, and external disturbances, respectively. Furthermore, Table 2 gives the trajectory tracking root-mean-square error (RMSE) of three controllers. In Figure 3, three control methods fulfill trajectory tracking errors gradually converge to zero. However, the TSMC and AFSMC have low robustness against the influences of actuator deadzones and faults that occur in *t* = 8 s. The proposed ANFTSMC provides much better robustness and transient response than TSMC and AFSMC, despite the influences of actuator deadzones and faults occur in *t* = 8 s. Particularly, according to Table 2, it can be observed that the proposed ANFTSMC scheme gives better trajectory tracking accuracy than TSMC and AFSMC methods. Furthermore, Figures 4 and 5 show the approximation performance of the proposed ANFTSMC scheme using FAT and DO. As shown by the results in Figure 4, it is obvious that the ANFTSMC using FAT offers a high estimation precision than AFSMC using FLS. In addition, it can be observed from Figure 5 that the proposed DO has good disturbance estimation capability. Therefore, it is concluded that the proposed ANFTSMC scheme provides a better tracking performance than TSMC and AFSMC methods of the continuum robot with actuator deadzones, actuator faults, and external disturbances.

## 5. Experiment Results

**Figure 6:**
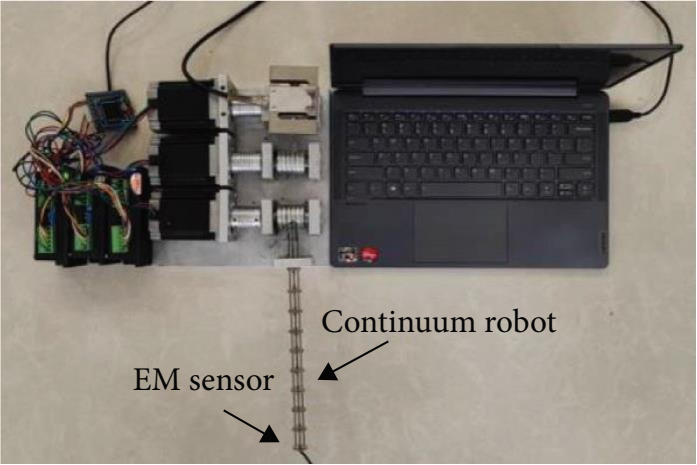
Experiment setup of the continuum robot.

To further indicate the effectiveness of the proposed ANFTSMC scheme, the experimental validations for the proposed controller on a continuum robotic system are illustrate in Figure 6 [5]. The physical parameters of the continuum robot are *L* = 210 mm and *r* = 5 mm. The electromagnetic (EM) tracking system is used to gauge the angle *θ* and *φ*. Furthermore, the controller parameters are the same as description in simulation. In the following experiments, we validate the tracking performance of the proposed ANFTSMC scheme in the presence and absence of the actuator deadzones and faults, respectively.

### 5.1. Case 1: Experiments with Actuator Deadzones and Faults

**Figure 7:**
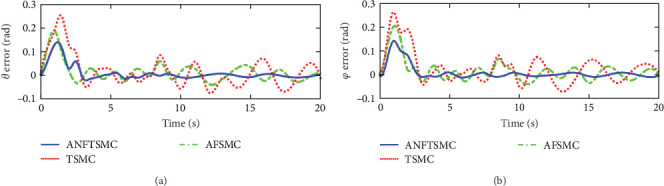
Experiments of the tracking errors in case 1: (a) tracking errors of angle *θ* and (b) tracking errors of angle *φ*.

**Figure 8:**
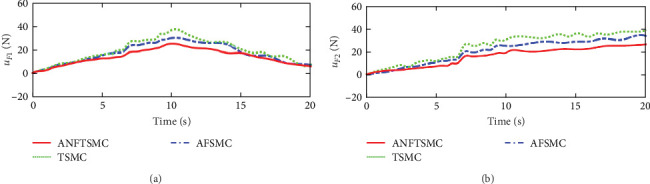
Experiments of the driving forces in case 1: (a) driving force *u*_*F*1_ and (b) driving force *u*_*F*2_.

Figures 7 and 8 illustrate the experiment results of the tracking errors and control inputs for the continuum robot in the existence of the actuator deadzones, actuator faults, and external disturbances, respectively. In addition, Table 3 provides the trajectory tracking RMSE of three controllers. Clearly, three controllers ensure that the tracking errors gradually converge to zero. From the outcomes viewed in Figure 7 and Table 3, we can see that the TSMC and AFSMC methods provide poor tracking performance for the continuum robot in the event of the actuator deadzones and faults. Particularly, the continuum robot instantly becomes instability when the actuator deadzones and faults occur in *t* = 8 s. Compared with TSMC and AFSMC methods, the proposed ANFTSMC scheme provides faster transient convergence speed, low tracking errors, and better robustness to resist the influences when the actuator deadzones and faults occur in *t* = 8 s. Furthermore, as shown in Figure 8, the control force inputs of the proposed ANFTSMC scheme provide a smooth control effect of the continuum robot than TSMC and AFSMC methods. Thus, it can be concluded that the proposed ANFTSMC scheme provides better tracking performance and robustness compared with other control methods, such as TSMC, and AFSMC in the existence of the actuator deadzones, actuator faults, and external disturbances.

### 5.2. Case 2: Experiments without Actuator Deadzones and Faults

In this subsection, we evaluate the tracking performance of the proposed ANFTSMC scheme for the continuum robot under external disturbances in absence of the actuator deadzones and faults.

**Figure 9:**
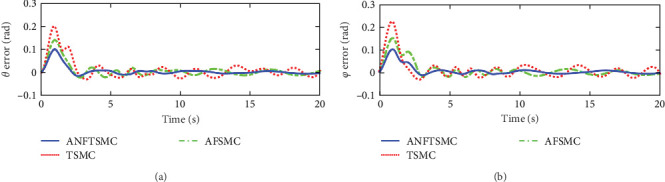
Experiments of the tracking errors in case 2: (a) tracking errors of angle *θ* and (b) tracking errors of angle *φ*.

**Figure 10:**
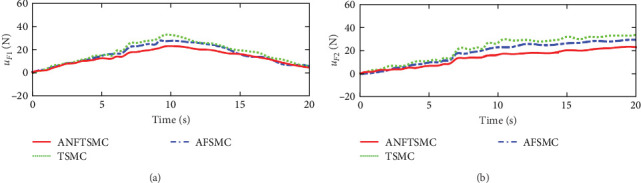
Experiments of the driving forces in case 2: (a) driving force *u*_*F*1_ and (b) driving force *u*_*F*2_.

The experiment results of the tracking errors and control inputs for the continuum robot under external disturbances without actuator deadzones and faults are shown in Figures 9 and 10, respectively.  Table 4 gives the tracking RMSE of three controllers. In Figure 9 and Table 4, it can be seen that the proposed ANFTSMC gives better tracking accuracy than TSMC and AFSMC under external disturbances. Thus, on the basis of the above experiment results, it is obtained that compared with TSMC and AFSMC methods, the proposed ANFTSMC scheme provides a good tracking performance of the continuum robot against external disturbances without actuator deadzones and faults.

## 6. Conclusions

In this paper, we proposed an ANFTSMC scheme combined with FAT and DO for the trajectory tracking of the continuum robot under effects of uncertain dynamics, unknown external disturbances, and unknown actuator deadzones/faults concurrently. A FAT is utilized to estimate the unknown dynamics and actuator deadzones/faults effectively. In addition, a DO is constructed to attenuate the influences of unknown external disturbances. Then, an ANFTSMC scheme using FAT and DO is developed, such that the continuum robot has good tracking precision. The proposed ANFTSMC scheme can maintain the merits of FTSMC, FAT, and DO. Afterward, the simulation studies show that the proposed ANFTSMC scheme for the continuum robot is effective compared to other advanced control approaches. Finally, the experiment results indicate that the proposed controller provides high tracking precision of the continuum robot facing system uncertainties, external disturbance, and actuator deadzones/faults.

In the future works, we will investigate the influences of the sensor faults in controller design of the continuum robot. Furthermore, future work will focus on developing an adaptive fault tolerant sliding-mode tracking controller with input and output constraints, to tackle the uncertainties, actuator deadzones, actuator faults, and sensor faults of the continuum robot systems.

## Figures and Tables

**Table 1:** Parameters of the controllers in simulations.

Controllers	Parameters
TSMC	$\begin{array}{l} K_{\sigma }=\text{diag}\left[8,8\right],K_{\eta }=\text{diag}\left[15,15\right],\lambda_{a}=0.8,\\ p_{a}=1.3,q_{a}=0.6 \end{array}$

AFSMC	$\begin{array}{l} K_{\beta }=\text{diag}\left[5,5\right],K_{\omega }=\text{diag}\left[12,12\right],h_{0}=0.7,\\ \hbar_{1}=0.5,\hbar_{2}=1.2,\xi_{M}=0.8,\xi_{R}=0.6 \end{array}$

ANFTSMC	$\begin{array}{l} K_{a}=\text{diag}\left[10,10\right],K_{0}=\text{diag}\left[6,6\right],K_{1}=\text{diag}\left[8,8\right],\\ K_{2}=\text{diag}\left[5,5\right],p_{1}=5,q_{1}=3,\lambda_{1}=2,\Gamma_{1}=1,\\ \Gamma_{2}=2,\xi_{1}=0.1,\xi_{2}=0.3,\Lambda_{1}=3,\Lambda_{2}=5,\\ \eta_{1}=0.5,\eta_{2}=0.3,\beta =5 \end{array}$

**Table 2:** Tracking errors of simulations.

Controllers	*θ* RMSE (rad)	*φ* RMSE (rad)
TSMC	0.0237	0.0283
AFSMC	0.0193	0.0172
ANFTSMC	0.0115	0.0128

**Table 3:** Tracking errors in case 1 of experiments.

Controllers	*θ* RMSE (rad)	*φ* RMSE (rad)
TSMC	0.0263	0.0307
AFSMC	0.0205	0.0192
ANFTSMC	0.0121	0.0139

**Table 4:** Tracking errors in case 2 of experiments.

Controllers	*θ* RMSE (rad)	*φ* RMSE (rad)
TSMC	0.0245	0.0291
AFSMC	0.0197	0.0183
ANFTSMC	0.0119	0.0132

## Data Availability

Data supporting this research article are available from the corresponding author upon reasonable request.

## Appendix

Proof First, considering the Lyapunov function *V*_1_ as follows:

(A.1) $$\begin{array}{c} V_{1}=\frac{1}{2}e_{1}^{T}e_{1}+\frac{1}{2}s^{T}M_{0}\left(x_{1}\right)s. \end{array}$$

Taking the derivative of Equation (A.1) combined with Equations (22), (23), (25), and Property 1, one has the following equation:

(A.2) $$\begin{array}{c} \begin{array}{c} {\dot{V}}_{1}=e_{1}^{T}{\dot{e}}_{1}+s^{T}M_{0}\left(x_{1}\right)\dot{s}+\frac{1}{2}s^{T}{\dot{M}}_{0}\left(x_{1}\right)s\\ =e_{1}^{T}\left(-K_{a}e_{1}+e_{2}\right)+s^{T}M_{0}\left(x_{1}\right)\left(K_{0}{\dot{e}}_{1}+K_{1}\lambda_{1}{\left[e_{1}\right]}^{\lambda_{1}-1}{\dot{e}}_{1}\right)\\ +s^{T}M_{0}\left(x_{1}\right)K_{2}\frac{p_{1}}{q_{1}}{\left[e_{2}\right]}^{\left(p_{1}/q_{1}-1\right)}{\dot{e}}_{2}+s^{T}C_{0}\left(x_{1},x_{2}\right)s\\ =e_{1}^{T}\left(-K_{a}e_{1}+e_{2}\right)+s^{T}M_{0}\left(x_{1}\right)\left(K_{0}{\dot{e}}_{1}+K_{1}\lambda_{1}{\left[e_{1}\right]}^{\lambda_{1}-1}{\dot{e}}_{1}\right)\\ +s^{T}C_{0}\left(x_{1},x_{2}\right)s+s^{T}K_{2}\frac{p_{1}}{q_{1}}{\left[e_{2}\right]}^{\left(p_{1}/q_{1}-1\right)}\left(u-C_{0}\left(x_{1},x_{2}\right)x_{2}\right.\\ \left.-G_{0}\left(x_{1}\right)-\gamma \left(x_{1},x_{2},u\right)-d-M_{0}\left(x_{1}\right){\dot{\mu }}_{1}\right). \end{array} \end{array}$$

Then, choosing Lyapunov function *V*_2_ as follows:

(A.3) $$\begin{array}{c} V_{2}=V_{1}+\frac{1}{2}{\tilde{\delta }}^{T}\Lambda^{-1}\tilde{\delta }+\frac{1}{2}\sum_{i=1}^{2}{\tilde{w}}_{i}^{T}\Gamma_{i}^{-1}{\tilde{w}}_{i}, \end{array}$$

where Λ = diag[Λ_1_, Λ_2_] expresses a positive definite matrix, and weight error ${\tilde{w}}_{i}={\hat{w}}_{i}-w_{i}^{*}$. Differentiating *V*_2_, one has the following equation:

(A.4) $$\begin{array}{c} V_{2}={\dot{V}}_{1}+{\tilde{\delta }}^{T}\Lambda^{-1}\dot{\tilde{\delta }}+\sum_{i=1}^{2}{\tilde{w}}_{i}^{T}\Gamma_{i}^{-1}{\dot{\tilde{w}}}_{i}. \end{array}$$

Substituting Equations (A.2) into Equation (A.4) yields the following equation:

(A.5) $$\begin{array}{c} \begin{array}{c} {\dot{V}}_{2}=e_{1}^{T}\left(-K_{a}e_{1}+e_{2}\right)+s^{T}M_{0}\left(x_{1}\right)\left(K_{0}{\dot{e}}_{1}+K_{1}\lambda_{1}{\left[e_{1}\right]}^{\lambda_{1}-1}{\dot{e}}_{1}\right)\\ +s^{T}C_{0}\left(x_{1},x_{2}\right)s+s^{T}K_{2}\frac{p_{1}}{q_{1}}{\left[e_{2}\right]}^{\left(p_{1}/q_{1}-1\right)}\left(u-C_{0}\left(x_{1},x_{2}\right)x_{2}\right.\\ \left.-G_{0}\left(x_{1}\right)-\gamma \left(x_{1},x_{2},u\right)-\delta -M_{0}\left(x_{1}\right){\dot{\mu }}_{1}\right)\\ +{\tilde{\delta }}^{T}\Lambda^{-1}\dot{\hat{\delta }}-{\tilde{\delta }}^{T}\Lambda^{-1}\dot{\delta }+\sum_{i=1}^{2}{\tilde{w}}_{i}^{T}\Gamma_{i}^{-1}{\dot{\hat{w}}}_{i}. \end{array} \end{array}$$

Substituting Equation (26) into Equation (A.5) and combined with Equation (28), one has the following equation:

(A.6) $$\begin{array}{c} \begin{array}{c} {\dot{V}}_{2}=-e_{1}^{T}K_{a}e_{1}-e_{1}^{T}e_{2}+s^{T}M_{0}\left(x_{1}\right)\left(K_{0}{\dot{e}}_{1}+K_{1}\lambda_{1}{\left[e_{1}\right]}^{\lambda_{1}-1}{\dot{e}}_{1}\right)\\ +s^{T}C_{0}\left(x_{1},x_{2}\right)s+s^{T}K_{2}\frac{p_{1}}{q_{1}}{\left[e_{2}\right]}^{\left(p_{1}/q_{1}-1\right)}\left(u-C_{0}\left(x_{1},x_{2}\right)x_{2}\right.\\ \left.-G_{0}\left(x_{1}\right)-W^{*T}\varphi \left(Z\right)-\varepsilon_{Z}+\tilde{\delta }-\hat{\delta }-M_{0}\left(x_{1}\right){\dot{\mu }}_{1}\right)\\ +{\tilde{\delta }}^{T}\Lambda^{-1}\dot{\hat{\delta }}-{\tilde{\delta }}^{T}\Lambda^{-1}\dot{\delta }+\sum_{i=1}^{2}{\tilde{w}}_{i}^{T}\Gamma_{i}^{-1}{\dot{\hat{w}}}_{i}. \end{array} \end{array}$$

Substituting Equations (31)–(33) into Equation (A.6), yields the following equation:

(A.7) $$\begin{array}{c} \begin{array}{c} {\dot{V}}_{2}\leq -e_{1}^{T}K_{a}e_{1}-\beta {\left\|s\right\|}^{2}+s^{T}K_{2}\frac{p_{1}}{q_{1}}{\left[e_{2}\right]}^{\left(p_{1}/q_{1}-1\right)}\left(W^{*T}\varphi \left(Z\right)+\tilde{\delta }-\varepsilon \right)\\ +{\tilde{\delta }}^{T}\Lambda^{-1}\dot{\hat{\delta }}-{\tilde{\delta }}^{T}\Lambda^{-1}\dot{\delta }+\sum_{i=1}^{2}{\tilde{w}}_{i}^{T}\Gamma_{i}^{-1}{\dot{\hat{w}}}_{i}. \end{array} \end{array}$$

Afterward, substituting Equations (27) and (30) into Equation (A.7), one has the following equation:

(A.8) $$\begin{array}{c} \begin{array}{ccc} {\dot{V}}_{2} & \leq & -e_{1}^{T}K_{a}e_{1}-\beta {\left\|s\right\|}^{2}+{\tilde{\delta }}^{T}\Lambda^{-1}h_{1}-s^{T}K_{2}\frac{p_{1}}{q_{1}}{\left[e_{2}\right]}^{\left(p_{1}/q_{1}-1\right)}\varepsilon \\ \; & + & \sum_{i=1}^{2}\delta_{i}^{T}\eta_{i}{\hat{\delta }}_{i}-\sum_{i=1}^{2}{\tilde{w}}_{i}^{T}\xi_{i}{\hat{w}}_{i}\\ \; & \leq & -e_{1}^{T}K_{a}e_{1}-\beta {\left\|s\right\|}^{2}+\frac{1}{2}\sum_{i=1}^{2}{\tilde{\delta }}_{i}^{T}\left(\Lambda_{i}^{-1}-\eta_{i}\right){\tilde{\delta }}_{i}+\frac{1}{2}\sum_{i=1}^{2}\Lambda_{i}^{-1}h_{1i}^{2}\\ \; & + & \frac{1}{2}\sum_{i=1}^{2}\eta_{i}b_{1i}^{2}-s^{T}K_{2}\frac{p_{1}}{q_{1}}{\left[e_{2}\right]}^{\left(p_{1}/q_{1}-1\right)}\varepsilon -\sum_{i=1}^{2}{\tilde{w}}_{i}^{T}\xi_{i}{\hat{w}}_{i}\\ \; & \leq & -e_{1}^{T}K_{a}e_{1}-s^{T}\left(\beta I-\frac{p_{1}}{2q_{1}}K_{2}\right)s-\frac{1}{2}\sum_{i=1}^{2}{\tilde{\delta }}_{i}^{T}\left(\eta_{i}-\Lambda_{i}^{-1}\right){\tilde{\delta }}_{i}+\frac{1}{2}\sum_{i=1}^{2}\Lambda_{i}^{-1}h_{1i}^{2}\\ \; & + & \frac{1}{2}\sum_{i=1}^{2}\eta_{i}b_{1i}^{2}+\frac{p_{1}}{2q_{1}}{\left({\left[e_{2}\right]}^{\left(p_{1}/q_{1}-1\right)}\varepsilon \right)}^{T}K_{2}\left({\left[e_{2}\right]}^{\left(p_{1}/q_{1}-1\right)}\varepsilon \right)-\sum_{i=1}^{2}{\tilde{w}}_{i}^{T}\xi_{i}{\hat{w}}_{i}\\ \; & \leq & -e_{1}^{T}K_{a}e_{1}-s^{T}\left(\beta I-\frac{p_{1}}{2q_{1}}K_{2}\right)s-\frac{1}{2}\sum_{i=1}^{2}{\tilde{\delta }}_{i}^{T}\left(\eta_{i}-\Lambda_{i}^{-1}\right){\tilde{\delta }}_{i}-\frac{1}{2}\sum_{i=1}^{2}{\tilde{w}}_{i}^{T}\xi_{i}{\tilde{w}}_{i}\\ \; & + & \frac{1}{2}\sum_{i=1}^{2}\Lambda_{i}^{-1}h_{1i}^{2}+\frac{1}{2}\sum_{i=1}^{2}\eta_{i}b_{1i}^{2}+\frac{p_{1}}{2q_{1}}{\overset{―}{\varepsilon }}^{T}K_{2}\overset{―}{\varepsilon }+\frac{1}{2}\sum_{i=1}^{2}w_{i}^{*T}\xi_{i}w_{i}^{*}\\ \; & \leq & -a_{2}V_{2}+b_{2} \end{array}. \end{array}$$

where $\overset{―}{\varepsilon }={\left[e_{2}\right]}^{\left(p_{1}/q_{1}-1\right)}\varepsilon ,a_{2}$ and *b*_2_ are two positive constants given by the following equations:

(A.9) $$\begin{array}{c} a_{2}=\min\left\{2\lambda_{\min}\left(K_{a}\right),\frac{2\lambda_{\min}\left(\beta I-\left(p_{1}/2q_{1}\right)K_{2}\right)}{\lambda_{\max}\left(M_{0}\left(x_{1}\right)\right)},|\min_{i=1,2}\frac{\eta_{i}-\Lambda_{i}^{-1}}{\lambda_{\max}\left(\Lambda^{-1}\right)},\min_{i=1,2}\frac{\xi_{i}}{\lambda_{\max}\left(\Gamma^{-1}\right)}\right\}, \end{array}$$

(A.10) $$\begin{array}{c} b_{2}=\frac{1}{2}\sum_{i=1}^{2}\Lambda_{i}^{-1}h_{1i}^{2}+\frac{1}{2}\sum_{i=1}^{2}\eta_{i}b_{1i}^{2}+\frac{p_{1}}{2q_{1}}{\overset{―}{\varepsilon }}^{T}K_{2}\overset{―}{\varepsilon }+\frac{1}{2}\sum_{i=1}^{2}w_{i}^{*T}\xi_{i}w_{i}^{*}. \end{array}$$

Furthermore, to assure *a*_2_ > 0, the parameters of *β*, *η*_*i*_, Λ_*i*_, and matrix *K*_2_ are selected to satisfy the following equations:

(A.11) $$\begin{array}{c} \lambda_{\min}\left(\beta I-\frac{p_{1}}{2q_{1}}K_{2}\right)>0, \end{array}$$

(A.12) $$\begin{array}{c} \min_{i=1,2}\left(\eta_{i}-\Lambda_{i}^{-1}\right)>0. \end{array}$$

Multiplying both sides of Equation (A.8) by *e*^*a*_2_*t*^, and integrating over [0, *t*], obtains the following equation:

(A.13) $$\begin{array}{c} \begin{array}{ccc} V_{2} & \leq & \left(V_{2}\left(0\right)-\frac{b_{2}}{a_{2}}\right)e^{-a_{2}t}+\frac{b_{2}}{a_{2}}\\ \; & \leq & V_{2}\left(0\right)+\frac{b_{2}}{a_{2}}\\ \; & \leq & \frac{1}{2}D_{1} \end{array}, \end{array}$$

where *D*_1_ = 2(*V*_2_(0) + *b*_2_/*a*_2_). Then, on the basis of Equation (A.12), we have the following equations:

(A.14) $$\begin{array}{c} \left\|e_{1}\right\|<\sqrt{D_{1}}, \end{array}$$

(A.15) $$\begin{array}{c} \left\|s\right\|<\sqrt{\frac{D_{1}}{\lambda_{\min}\left(M_{0}\left(x_{1}\right)\right)}}, \end{array}$$

(A.16) $$\begin{array}{c} \left\|\tilde{\delta }\right\|<\sqrt{\frac{D_{1}}{\lambda_{\min}\left(\Lambda^{-1}\right)}}, \end{array}$$

(A.17) $$\begin{array}{c} \left\|\tilde{W}\right\|<\sqrt{\frac{D_{1}}{\lambda_{\min}\left(\Gamma^{-1}\right)}}. \end{array}$$

Therefore, according to Equations (A.14)–(A.17), it can be obtained that the errors $e_{1},s,\tilde{\delta }$, and $\tilde{W}$ are uniformly bounded and gradually converge to zero. This completes the proof.
